# Design of a CANFD to SOME/IP Gateway Considering Security for In-Vehicle Networks

**DOI:** 10.3390/s21237917

**Published:** 2021-11-27

**Authors:** Zheng Zuo, Shichun Yang, Bin Ma, Bosong Zou, Yaoguang Cao, Qiangwei Li, Sida Zhou, Jichong Li

**Affiliations:** 1 School of Transportation Science and Engineering, Beihang University, Beijing 102206, China; zuozuo@buaa.edu.cn (Z.Z.); yangshichun@buaa.edu.cn (S.Y.); caoyaoguang@buaa.edu.cn (Y.C.); helloworld_li@buaa.edu.cn (Q.L.); zhousida@buaa.edu.cn (S.Z.); ZY1913121@buaa.edu.cn (J.L.); 2 China Software Testing Center, Beijing 100038, China; zoubosong@cstc.org.cn

**Keywords:** vehicle, CAN/CANFD, Ethernet, SOME/IP, gateway, security, MAC, AEAD

## Abstract

In recent years, Ethernet has been introduced into vehicular networks to cope with the increasing demand for bandwidth and complexity in communication networks. To exchange data between controller area network (CAN) and Ethernet, a gateway system is required to provide a communication interface. Additionally, the existence of networked devices exposes automobiles to cyber security threats. Against this background, a gateway for CAN/CAN with flexible data-rate (CANFD) to scalable service-oriented middleware over IP (SOME/IP) protocol conversion is designed, and security schemes are implemented in the routing process to provide integrity and confidentiality protections. Based on NXP-S32G, the designed gateway is implemented and evaluated. Under most operating conditions, the CPU and the RAM usage are less than 5% and 20 MB, respectively. Devices running a Linux operating system can easily bear such a system resource overhead. The latency caused by the security scheme accounts for about 25% of the entire protocol conversion latency. Considering the security protection provided by the security scheme, this overhead is worthwhile. The results show that the designed gateway can ensure a CAN/CANFD to SOME/IP protocol conversion with a low system resource overhead and a low latency while effectively resisting hacker attacks such as frame forgery, tampering, and sniffing.

## 1. Introduction

The traditional automotive electrical/electronic architecture (EEA) is a distributed control architecture that has been applied for decades with a small number of electronic control units (ECUs). For example, the Audi A8 had only five ECUs in 1993. However, with the continuous upgrading of vehicle electronics, the number of ECUs in a vehicle has experienced an enormous increase. Some luxury cars now have even more than 100 ECUs [1]. The rapid increase in the number of ECUs has not only led to an increase in the wiring harness and assembly costs but has also prevented automobiles from completing rapid iterations and thereby keeping pace with the development of information technology. Recently, pioneering vehicle companies such as Tesla have taken the lead in launching domain-centralized EEA, proving its huge potential for reducing vehicle manufacturing costs, promoting the development of autonomous driving and vehicle-to-X (V2X), etc. Although the major manufacturers incorporate different design details, few have questioned the developing trend of EEA with the core concept of centralization across the domain and entire vehicle [2].

Domain-centralized and vehicle-centralized EEA can be used to construct a new in-vehicle communication environment that makes use of the automotive Ethernet as a core backbone. This network can provide a higher communication bandwidth for the communication traffic required by V2X; sensors such as cameras and lidars, which are required for intelligent driving; and the human-computer interaction required by the in-vehicle infotainment (IVI) system. However, traditional buses, such as CAN and local interconnect network (LIN), are not only cost-efficient, well-tested, and robust but can also provide sufficient bandwidth for many low-end applications. Diverse networks should be used in automobiles in order to provide different combinations of performance, cost, and features. Thus, automotive Ethernet will dominate the in-vehicle network together with traditional buses (CAN, LIN, and FlexRay) in the foreseeable future [3].

In-vehicle gateway devices can provide effective support for the practical problems that arise from the long-term coexistence of multiple protocols and rapid increases in communication traffic. The gateway must provide a seamless routing between traditional protocols, such as CAN, CANFD, LIN, and FlexRay, and the Ethernet protocol [4,5,6,7,8,9,10,11,12]. For example, in an advanced driving assistance system (ADAS), the controller not only needs to obtain perceptual data from the lidar sensor through gigabit Ethernet but also needs to obtain the vehicle speed information and other types of data via the CAN bus.

The rapid development of intelligent connected vehicles (ICV) has not only created the new requirements for the innovations in automobile EEA but also led to worsened cyber security issues compared with those seen in the traditional Internet industry [13,14,15]. On the one hand, traditional automobiles are defined as a closed system, and security protection against external threats is not considered at the beginning of the design process. However, the ICV needs to connect with the open network and interact with other objectives in the traffic; at the same time, it must be able to face the cyber security issues outside. On the other hand, automobile cyber security issues are more serious than computer cyber security issues. If the ICV is attacked, it will not only cause the leakage of users’ personal privacy data but will also directly threaten the life and property safety of drivers and passengers.

Therefore, for a new in-vehicle network where Ethernet and the traditional CAN bus coexist, this paper proposes and implements a CAN/CANFD to SOME/IP gateway system with cyber security protection mechanisms. The main contributions of this paper include:

1. The proposal of an in-vehicle gateway system for CAN/CANFD to SOME/IP protocol conversion. This system is composed of five modules, including a SOME/IP receive module, a CAN receive module, a protocol transform module, a security module, and a SOME/IP send module. The execution logic flow is as follows: first, the service request (request message) from the domain controller (e.g., ADAS) is received through the SOME/IP protocol. Then, the information required by the domain controller is obtained from the CAN bus through the CAN/CANFD protocol and converted into the SOME/IP message format. Finally, the SOME/IP protocol is used to return data (response message) to the domain controller;
2. Providing three strengths of cyber security protection mechanisms in the process of the gateway system protocol conversion. The application developers can tune them according to security protection strength requirements to achieve the best compromise between security and performance. Among them, Scheme 1 is implemented based on the MAC algorithm to provide integrity protection for the routing process. It can resist frame forgery and frame tampering attacks. Scheme 2 is implemented based on the AEAD algorithm to provide integrity and confidentiality protection for the routing process. It can resist frame forgery, frame tampering, and frame sniffing attacks. However, Scheme 3 is not provided with cyber security protection in order to obtain a higher routing performance;
3. Building the experimental platform, including the gateway, ADAS, and CAN/CANFD network and evaluating the protocol conversion and cyber security protection performance of the designed gateway. The experiment is carried out with the CAN/CANFD protocol type, CAN/CANFD payload length, and safety mechanism type as variables. At the same time, the experiment conducts a detailed evaluation of the gateway performance based on four indicators: CPU usage, memory usage, latency, and the percentage of the effective load of the SOME/IP message sent by the gateway program.

The rest of this paper is organized as follows: Section 2 reviews the related work in this field. Section 3 introduces the preliminary knowledge related to this paper. Section 4 describes the designed gateway system in detail. Section 5 quantitatively evaluates the performance indicators of the designed gateway system based on experiments. Section 6 summarizes the full text and draws conclusions.

## 2. Related Work

In this section, we review the existing literature available on routing designs and authentication techniques for in-vehicle networks and the development of automotive Ethernet.

### 2.1. Vehicle Routing

**Figure 1:**
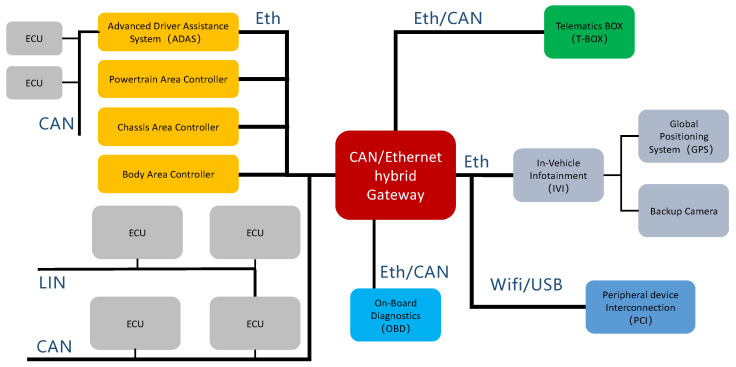
Example of an automotive hybrid gateway and domain controller topology.

The communication of ECUs using different protocols in the vehicle heterogeneous network requires the support of the routing mechanism. Unlike the routing mechanism in the Internet field, which is divided into multiple layers, the in-vehicle network routing mechanism usually contains only one or two layers, and its main tasks are unpacking messages, packaging messages, and finding source and destination addresses to support mutual communication between ECUs [16]. The executive unit of the routing mechanism is usually an in-vehicle gateway or a domain controller that maintains a routing table, including the CAN-ID, IP address, transmission priority, and other information. According to the advanced nature of the vehicle network architecture, three types of gateways exist in the vehicle network. These are the traditional CAN gateway using the CAN network as the backbone, the Ethernet gateway with Ethernet as the backbone network, and the CAN/Ethernet hybrid gateway. An example of a hybrid gateway and domain controller topology is shown in Figure 1.

In recent years, with the development of automotive EEA, the number of studies on in-vehicle gateways and routing mechanisms has gradually increased [4,5,6,7,8,9,10,11,12]. Trong Yen Lee et al. propose a routing mechanism between Ethernet and FlexRay. This mechanism is to be integrated into a gateway system built with FPGA in [4,5]; cyber security protection is created by adding message authentication to the routing process. The results show that the FPGA-based gateway system has suitable latency and power consumption characteristics. Jin Seo Park et al. propose a routing method between Ethernet and CAN/CANFD in [6,7]. The routing method is divided into a direct routing mechanism and an indirect routing mechanism according to the integrated message authentication method in the routing process, and the routing performance is measured and evaluated. The results show that the transmission time of the CAN message from the ECU to the gateway accounts for the largest proportion of time taken in the entire routing process. However, the Ethernet ends of the routing mechanisms proposed by Trong Yen Lee et al. and Jin Seo Park et al. do not consider the possible performance impact caused by standardized vehicle application layer protocols such as SOME/IP. In addition, their cyber security protection mechanisms only contain a MAC to ensure message integrity without considering confidentiality protection. Kanchan Yadav et al. propose the use of an Ethernet to CAN gateway system in [11]. The network management software implemented is written in CAPL and consists of two independent parts: basic modules (send module, protocol transform module, and receive module) and configuration files (module configuration information and rules). However, their work does not involve any cyber security protection mechanism. ChangYoung Jo et al.propose the use of a multi-core gateway system for an Ethernet and CAN/CANFD hybrid network in [12]. The gateway has a multi-core architecture and is equipped with a new operating algorithm and scheduling algorithm. The simulation results based on the CANoe software show that the multi-core gateway they designed has a higher performance than the single-core gateway.

In addition, some scholars have already carried out research on related in-vehicle routing mechanisms, but most of these studies do not consider the possible impact of cyber security protection mechanisms and automotive Ethernet application layer protocol. In [8], the authors describe the design of the routing mechanism between CAN, FlexRay, and Ethernet. The authors of [9,10] describe the design of a routing mechanism between FlexRay and Ethernet; the paper [9] verifies the use of this mechanism in a gateway system built using FPGA. In Table 1, a comparison of the main features of the routing mechanisms proposed by previous studies and this paper is provided. Among these, the use of Ethernet (custom) shows that the author does not use a standardized application layer protocol, but directly uses TCP/UDP for data transmission and reception.

Compared with previous work, the CAN/CANFD to SOME/IP gateway system designed in this paper makes two main innovations. On the one hand, this system supports the SOME/IP protocol, which is considered to be a very promising service-oriented architecture (SOA) middleware for vehicles. On the other hand, three different strengths of cyber security protection mechanisms are provided in the gateway protocol conversion process, which can provide integrity and confidentiality cyber security protection for the data transmission process.

### 2.2. Authentication Techniques for In-Vehicle Networks

Automobile cyber security protection is a typical interdisciplinary topic. At present, most scholars in this field have backgrounds in computer networks, computer security, mathematics, control algorithms, and cryptography. Research on the cyber security of in-vehicle networks involves electronics, embedded systems, and even mechanical fields, and these contents often have a certain degree of heterogeneity in different vehicle models. Therefore, this mismatch of professional backgrounds renders the current research on in-vehicle cyber security relatively insufficient, and most related studies are conducted on the CAN bus [17,18,19,20,21,22,23]. Additionally, there have been few studies involving vehicle gateways and automotive Ethernet security [4,5,6,7,24,25].

Giampaolo et al. [17] propose the use of an algorithm named TOUCAN that implements AES128 and Chaskey MAC to provide integrity and confidentiality protection for CAN bus data transmission. The authors test the algorithm on the STM32F407 controller, and their results show that the total running time of the encryption and MAC is about 12 ms, with MAC occupying 3 bytes in 8 bytes of data. Agrawal et al. [18] propose a gateway to ensure the cyber security of the CANFD bus. The ECUs on the bus and the gateway need pre-installed private keys and public keys, respectively. The gateway not only needs to transmit messages between high- and low-speed CAN buses but is also responsible for the distribution of a session key to each ECU and for checking the freshness of the key. Carel et al. [19] use the lightweight Chaskey MAC algorithm to provide integrity protection for CANFD bus data transmission and add a 4-bytes message counter to the CANFD message to prevent possible replay attacks, but this method cannot guarantee confidentiality during data transmission. Marco et al. [24,25] designed a new security mechanism to ensure the integrity, non-repudiation, and confidentiality of SOME/IP message transmission. This mechanism includes authentication request and authentication response headers to the SOME/IP payload data and provides an authentication method with three security levels. After testing, the method is found to have a slight impact on the transmission rate of SOME/IP messages in in-vehicle networks.

## 3. Preliminary Background

This section briefly reviews the background knowledge related to the designed CAN/CANFD to SOME/IP gateway, including the CANFD protocol, SOME/IP protocol, and MAC and AEAD algorithms.

### 3.1. CANFD

**Figure 2:**
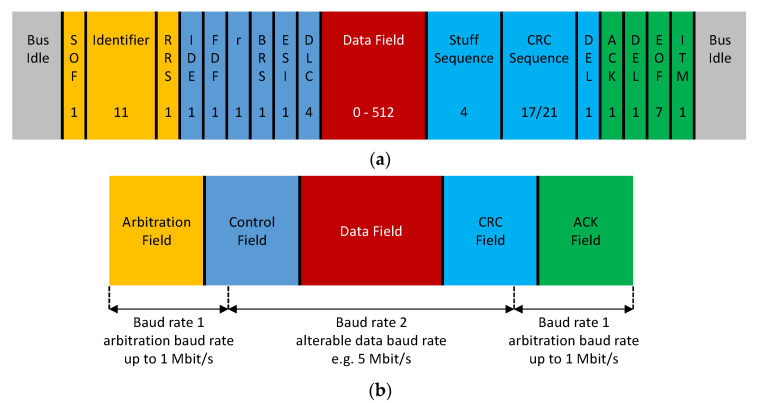
Frame structure and baud rate switch of CANFD: (**a**) CANFD frame structure with an 11-bit identifier; (**b**) baud rate switch of CANFD.

CANFD is a CAN replacement bus solution proposed by Bosch in 2011; it was included in the ISO 11898 series of international standards in 2015 [26]. Compared with CAN, CANFD makes two main contributions to increase the bandwidth of the automobile bus: a longer effective data segment (64 bytes) and a higher data transmission rate. When the CANFD data frame is transmitted, the arbitration field, part of the control field, part of the CRC field, and the ACK field use the standard CAN bus communication baud rate. The baud rate can be switched to a higher value when the data field is transmitted. The data transmission baud rate can be greater than 1 Mbit/s, reaching 5 Mbit/s or even higher. The CANFD standard frame structure and the switch of its transmission rate are shown in Figure 2.

### 3.2. Automotive Ethernet

The CAN bus is currently the most widely used in-vehicle bus protocol with the characteristics of low cost, high reliability, and real-time operation. However, with the development of the automotive EEA and the increase in the amount of interactive data, the demand for network bandwidth in automotive applications has shown explosive growth. Additionally, automotive Ethernet has become an important development direction for in-vehicle networks [27]. The development of automotive Ethernet relies heavily on the standardization promotion work of some alliances, such as IEEE, OPEN, AUTOSAR, and AVnu. Here, we focus on the development of relevant protocol standards.

**Figure 3:**
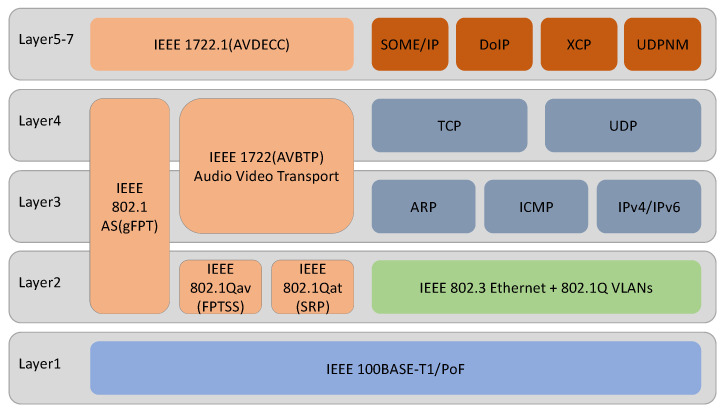
Automotive Ethernet and its upper-layer protocol architecture.

The current main automotive Ethernet and the upper-layer protocols supported by it are drawn in the ISO/OSI seven-layer architecture, as shown in Figure 3. There are three representative achievements in the physical layer of automotive Ethernet: the BroadR-Reach technology of Broadcom Corporation, the AVB/TSN technology of AVnu Alliance, and TTEthernet of TTTech, among which BroadR-Reach technology has been standardized as 100BASE-T1 by IEEE802.3bw, which is also called OABR (OPEN Alliance BroadR-Reach) [27]. Above the physical layer, some standard protocols such as IEEE 802.1AS, IEEE 802.1Qat, IEEE 802.1Qav, IEEE 1722 (AVBTP), and IEEE 1722.1 (AVDECC) [28,29,30,31,32,33] can be used to implement Ethernet AVB transmission. AVB enhances the real-time performance of traditional Ethernet audio and video transmission by adding precision clock synchronization and bandwidth reservation based on traditional Ethernet, which is a real-time audio and video transmission technology for IVI systems with significant development potential.

At the same time, automotive Ethernet adopts the IEEE 802.3 interface standard, which can seamlessly support the widely used TCP/IP protocol cluster without adaptation. The corresponding application layer protocols include SOME/IP, Do/IP, XCP, UDPNM, etc. Among these, SOME/IP is a scalable middleware that is used to transmit service information. It can be adapted to devices of different sizes, ranging from a small camera to an IVI system or an autopilot module. Compared with the traditional CAN bus, which is a signal-oriented communication method, SOME/IP is a service-oriented communication method [34]. DolP is a diagnostic transmission protocol based on Ethernet that can encapsulate UDS and transmit it based on the IP network [35,36]. XCP is mainly used for calibration, measurement, small-scale programming, and flashing [37]. UDPNM is a network management protocol based on automobile Ethernet developed by AUTOSAR, which can effectively realize the coordinated sleep and wake-up of automobile Ethernet nodes.

### 3.3. SOME/IP

**Figure 4:**
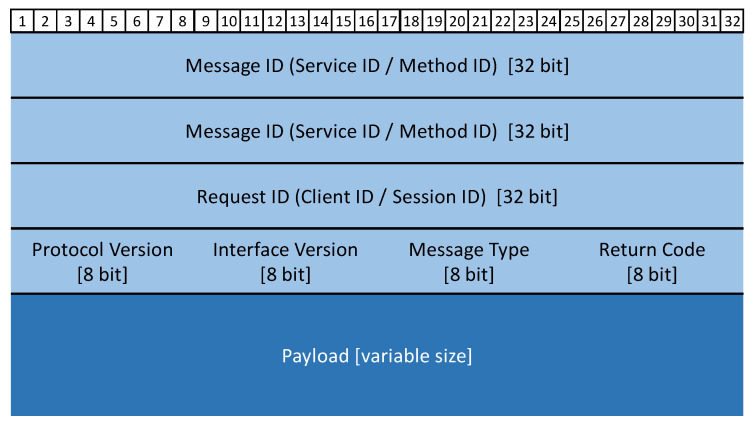
SOME/IP frame structure.

SOME/IP is one of the core protocols in automotive Ethernet technology and uses SOA software development logic to achieve isolation and modular design. It is located in layers 5–7 of the ISO/OSI seven-layer architecture and can use TCP or UDP as the transport layer protocol, as shown in Figure 2. SOME/IP was first proposed by the BMW Group in 2011. The AUTOSAR Alliance started to support the SOME/IP protocol from version 4.1 and completed the definition of the SOME/IP standard in version 4.3 [34]. The data frame structure is shown in Figure 4.

SOME/IP provides three major communication models. The first is service discovery (SD), which can dynamically inform about availability, access methods of service instances in in-vehicle communication, and manage subscriptions to selected services. The second is, remote procedure calls (RPC), which call remote service functions through request/response and read the return value. The third is the publish/subscribe mechanism, which decouples the sender and receiver of the message. Additionally, when an event occurs, the corresponding service publishes a new notification from which interested clients can obtain the corresponding data by subscribing to the event [38,39]. However, although SOME/IP is considered to be a very promising SOA middleware, it does not include any security function to protect applications and transmitted data from malicious attacks.

### 3.4. MAC and AE/AEAD

**Figure 5:**
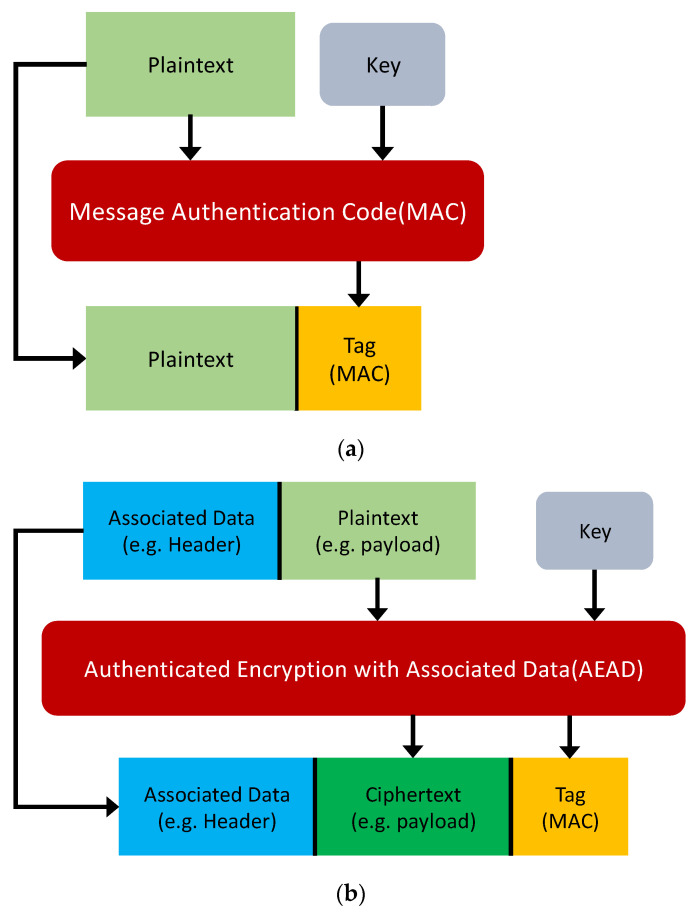
Working principle of the MAC and AEAD algorithms: (**a**) MAC; (**b**) AEAD.

Message authentication code (MAC), also known as cryptographic checksum, is a cryptographic authentication technology that mainly provides message integrity protection [40]. The working principle of the MAC algorithm is shown in Figure 5a. It uses the key to generate a fixed-length short data block that is attached to the back of the message and sends it to the receiver together. Assuming that communication parties A and B share the symmetric key K and that integrity protection is required by the transmitted message. A can therefore use the MAC algorithm to meet the requirements. The calculation formula for the MAC algorithm is shown in Formula (1):

(1) $$\mathrm{MAC}=f_{\mathrm{MAC}}(K,M),$$

In the formula, M is the input message, $f_{\mathrm{MAC}}$ is the MAC function, K is the shared symmetric key, and MAC is the message authentication code.

After receiving the message and MAC, the receiver uses the same key K to perform the same MAC calculation on the message to obtain a new MAC and compares the received MAC with the calculated MAC. If the received MAC is the same as the calculated MAC, the receiver can believe that the information has not been modified during transmission. Therefore, the message integrity authentication is completed.

Commonly used MAC algorithms include HMAC, CMAC, GMAC, Poly1305, etc. The two most widely used algorithms, HMAC and CMAC, are applied in the secure routing proposed in this paper.

AEAD (authenticated encryption with associated data) refers to the encryption system, which provides both confidentiality and integrity in communication [40]. Many applications and protocols require both forms of security guarantee, for example, a data packet containing the header and the payload. The header part is sent in plain text to provide the receiver with the information needed to parse the message. Its MAC needs to be calculated to provide integrity protection. The payload paopenlet rt is the valid data that needs to be transmitted; this must be encrypted, and the MAC must be calculated to provide integrity and confidentiality protection at the same time. The working principle of the AEAD algorithm is shown in Figure 5b. AEAD provides confidentiality (e.g., valid data) for the data packet to prevent unauthorized reading, modification, and forgery. At the same time, AEAD only provides integrity protection for non-confidential information (e.g., header), ensuring the network equipment can read data normally while preventing tampering or replacement [41]. Assuming that there are communication parties, A and B, that share the symmetric key K, and confidentiality and integrity protection are required by the transmitted message. A can use the AEAD algorithm to meet the requirements. The calculation formula for the AEAD algorithm is shown in Formula (2):

(2) $$[M_{CT},\mathrm{MAC}]=f_{\mathrm{AEAD}}(K,M_{AD},M_{PT}),$$

In this formula, $M_{AD}$ is the part of the input message that needs to be integrity protected, $M_{PT}$ is the part of the input message that needs to be protected by integrity and confidentiality, K is the shared symmetric key, $f_{\mathrm{AEAD}}$ is the AEAD function, MAC is the message authentication code, and $M_{CT}$ is encrypted $M_{PT}$.

After receiving the message and MAC, the receiver performs a reverse AEAD calculation on the message to obtain a new MAC and decrypted valid information. If the received MAC is the same as the calculated MAC, the receiver can believe that the information has not been modified during transmission; that is, it can believe that the effective information obtained by decryption has not been tampered with.

AEAD can be realized by simply combining the encryption algorithm and the authentication algorithm, but it is likely to cause security risks due to improper design [42]. Therefore, a scheme that can be used to achieve encryption and authentication at the same time has gradually appeared in the industry, including AES-GCM and Chacha20-Poly1305, which are involved in this paper.

## 4. Design of a CAN/CANFD to SOME/IP Gateway

### 4.1. Application Scenarios

**Figure 6:**
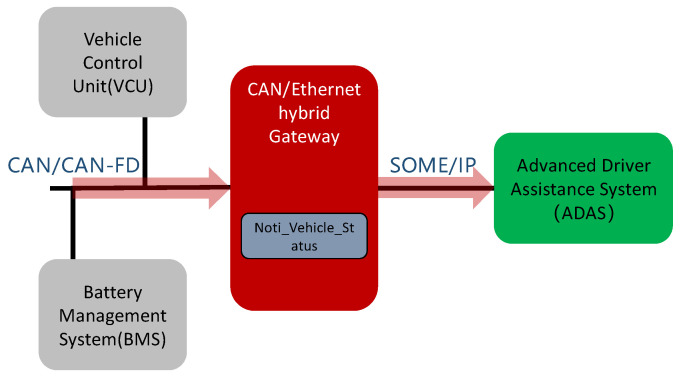
Application case of the CAN/CANFD to SOME/IP gateway.

Considering the business scenario shown in Figure 6, vehicle speed, battery status, faults, and other information from the vehicle control unit (VCU) or battery management system (BMS) must be reported to ADAS via a gateway to enable the latter to deliver functions, such as adaptive cruise control (ACC), intelligent speed assistance (ISA), and heads-up display (HUD). Among these, VCU and BMS exchange data with the gateway through CAN/CANFD, while ADAS exchanges data with the gateway through SOME/IP.

This scenario can be realized with the help of a CAN/CANFD to SOME/IP gateway. The SOME/IP protocol-based information-reporting service Noti_Vehicle_Status runs in the gateway. When ADAS requests the service Noti_Vehicle_Status, the gateway responds and encapsulates the CAN/CANFD messages from the CAN bus into a SOME/IP message before sending them to ADAS through the SOME/IP protocol.

### 4.2. Gateway Algorithm and System Architecture

Taking the scenario shown in Figure 6 as an example, the system structure and implementation process of the designed CAN/CANFD to SOME/IP gateways are described as follows.

The request-response communication model of the SOME/IP protocol is used in the interaction process between the gateway and ADAS. In this paper, vsomeip is used to implement this communication model. Vsomeip is a SOME/IP open-source implementation in the GENIVI project that is based on the Mozilla Public License v2.0 protocol and contributed by BMW [43].

**Figure 7:**
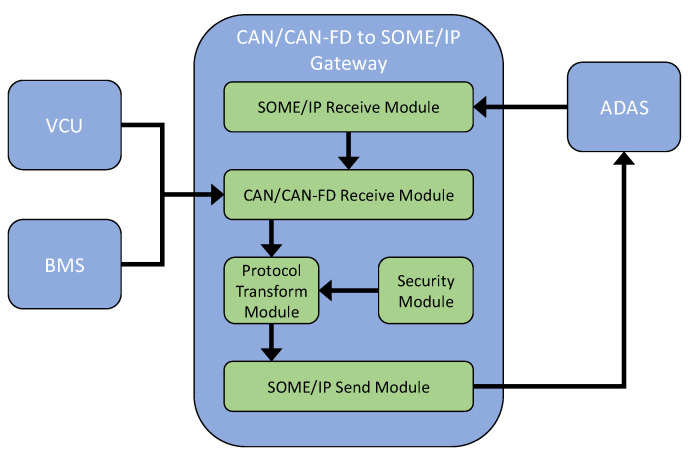
Structure of a CAN/CANFD to SOME/IP gateway system.

Running the application Server APP in the gateway: Server APP provides the information-reporting service Noti_Vehicle_Status, and its corresponding service instance is the Noti_Vehicle_Status_instance. The designed gateway function is carried out by this service. The service Noti_Vehicle_Status consists of five modules, as shown in Figure 7, including a SOME/IP receive module, a CAN receive module, a protocol transform module, a security module, and a SOME/IP send module. The SOME/IP receive module is responsible for receiving service requests from other ECUs. The CAN receive module is responsible for receiving CAN/CANFD messages from the CAN bus. The protocol transform module is responsible for encapsulating the valid data of CAN/CANFD messages into the frame of SOME/IP messages. The security module is responsible for completing cyber security functions, such as MAC calculation and data encryption, during the protocol transformation process. The SOME/IP send module is responsible for sending the encapsulated SOME/IP message to the service requester.

**Figure 8:**
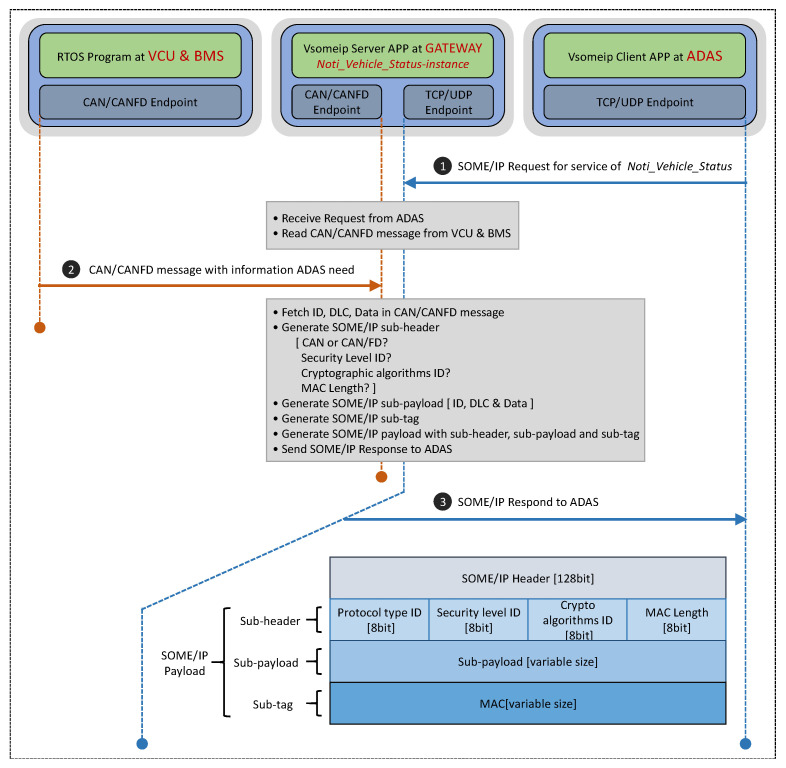
Workflow of the designed gateway.

The function realization process of the designed gateway is shown in Figure 8, which includes three message interactions.

Message 1:

Running the application Client App in the ADAS: the Client App sends the request message to the Server APP running in the gateway in order to request the service Noti_Vehicle_Status.

Message 2:

After receiving the request message, the service Noti_Vehicle_Status running in the gateway collects CAN/CANFD messages and extracts valid information such as CAN/CANFD ID, DLC, and data.

Message 3:

The service Noti_Vehicle_Status constructs a sub-header, sub-payload, and sub-tag in sequence then encapsulates these fields into a complete SOME/IP payload. Finally, it returns a response message to ADAS. Among these, the sub-header contains information, such as the CAN protocol type, security level, encryption algorithm, and MAC length, to inform the receiver that the information contained in this message comes from the CAN2.0 protocol or the CANFD protocol. The security mechanism is adopted in the transmission process. The sub-payload contains CAN/CANFD ID, DLC, data, and other information, meaning that the final receiver can parse these data. The sub-tag is the message authentication code.

The SOME/IP message structure finally generated by the service Noti_Vehicle_Status is shown in Figure 8. The specific security mechanism used in the process of message generation and transmission is described in the next section.

### 4.3. Security Mechanism

In the vehicle communication environment, common types of attacks [44] include frame forgery, frame tampering, frame sniffing, and DoS attacks. Among these, DoS attacks can be defended by firewall and intrusion detection technologies, and frame sniffing, frame forgery, and frame tampering can all be defended by cryptographic security protection technologies.

**Figure 9:**
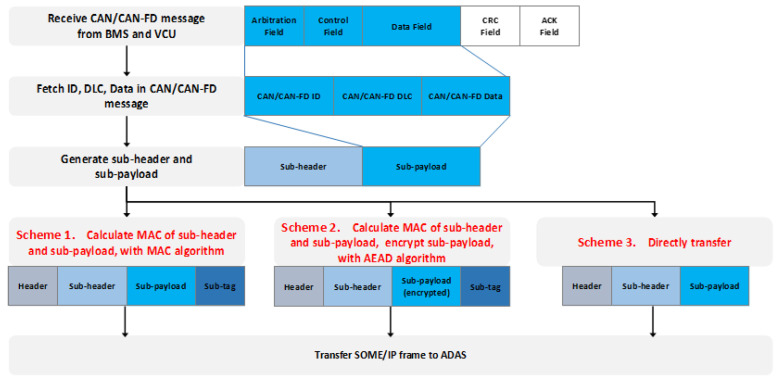
Security module flow chart and message composition at each stage.

The security module of the CAN/CANFD to SOME/IP gateway designed in this paper is used to ensure the security of data transmission from the gateway to ADAS. It is worth noting that this paper implements two security schemes with different strengths and adds direct forwarding (no security protection) as a control group. The security module’s flow chart and the message composition at each stage of the three routing schemes are shown in Figure 9.

- Scheme 1 uses the MAC algorithm to calculate the message authentication code of the sub-header and sub-payload as the sub-tags to provide integrity protection for the routing process and resist frame forgery and frame tampering attacks;
- Scheme 2 uses the AEAD algorithm to calculate the message authentication code of the sub-header and sub-payload as the sub-tag and encrypts the sub-payload, which provides integrity and confidentiality protection for the routing process and resists frame forgery and frame tampering, and frame sniffing attacks;
- Scheme 3 does not use security protection.

The MAC and AEAD algorithms used in the designed security module are shown in Table 2, while the values of the sub-header fields corresponding to different security schemes are shown in Table 3.

The principle of a hash-based message authentication code (HMAC) is to hash the key and the message together to obtain the message authentication code [45]. The HASH function it uses can be easily replaced according to the requirements of security strength: SHA256 is used here.

The principle of cipher-based message authentication code (CMAC) is to perform block encryption with the key and the message [46]. The block encryption function it uses can also be replaced according to the requirements of security strength, and here, AES128 is used.

AES-GCM uses the AES block encryption algorithm to achieve confidentiality protection and uses chained Galois field multiplication to achieve integrity protection. It has a high computational speed under the premise of an accelerated instruction set [47].

Chacha20-Poly1305 uses the Chacha20 stream encryption algorithm to achieve confidentiality protection and Poly1305 MAC to achieve integrity protection, and its software-based calculation speed is relatively high [48].

## 5. Implementation and Evaluation of the Designed Gateway

The main contribution of this paper is that a gateway is designed for CAN/CANFD to SOME/IP protocol conversion, while three security protection methods are implemented in the routing process to provide integrity and confidentiality protection for message conversion and transmission. The three security approaches are designed with different security strengths based on the MAC and AEAD algorithms. In order to evaluate the protocol conversion performance and the security mechanism performance of the designed gateway system, in this section, we implement the designed gateway based on an embedded system and experimentally evaluate its performance.

### 5.1. Hardware Environment

**Figure 10:**
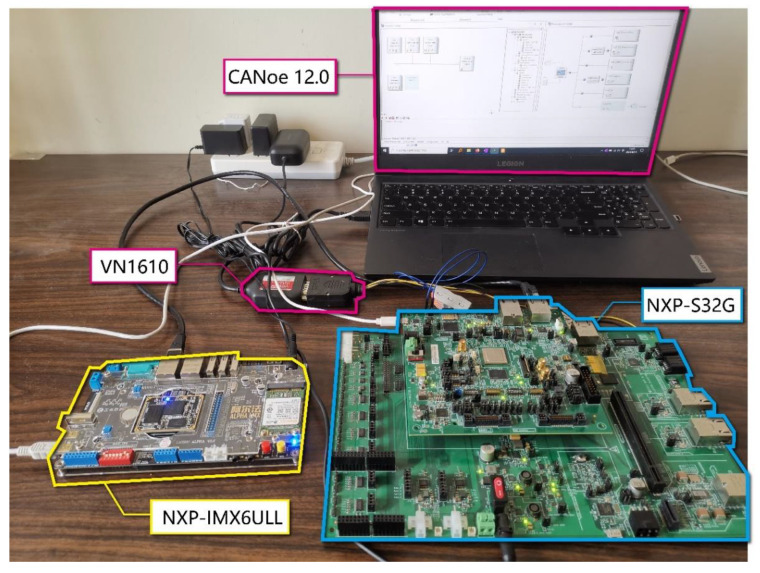
Experimental environment.

The experimental environment is built according to the application scenarios described in Section 4.1. The main components of the experiment are shown in Table 4, and the actual experimental environment is shown in Figure 10. The experimental environment is mainly composed of three parts: the gateway, ADAS, and CAN/CANFD bus, which are described as follows.

- Gateway

The NXP-S32G-274evb development board is used to realize the designed gateway system. The S32G chip is a multi-core heterogeneous architecture, equipped with 3 Arm Cortex-M7 cores and 4 Arm Cortex-A53 cores that supports the AES acceleration instruction set, 16 CANFD channels, and a 1000 Mbps Ethernet channel. Among these, A53 is clocked at 1000 MHz, and M7 is clocked at 400 MHz.

The main program of the gateway is implemented in the Linux 4.8 system carried in the A53 core, and its execution logic flow is shown in Figure 8. Firstly, the service request from ADAS is awaited, then the CAN/CANFD bus data are collected and converted after receiving the service request; finally, the converted data are returned to ADAS.

The request-response communication model of the SOME/IP protocol is used as an application layer protocol for the communication between the gateway and ADAS, while the UDP protocol is used as the transport layer protocol. The SOME/IP communication is developed based on vsomeip. The communication between the gateway and the CAN/CANFD bus is implemented based on the native CAN socket of the Linux system. The MAC and AEAD algorithms used in the gateway program are implemented based on OpenSSL. The OpenSSL library contains a very rich set of cryptographic algorithms that are widely used in both commercial and academic fields.

- ADAS

The NXP-IMX6ULL-alpha development board is used to implement the ADAS controller. The IMX6ULL is equipped with a clocked at 1000 MHZ Arm Cortex-A7 core and supports 2 CAN channels and a 100 Mbps Ethernet channel. In the experiment, the ADAS program does not contain ADAS-related applications and only exists as an Ethernet node. It is only responsible for sending a service request to the gateway and receiving the data returned by the gateway. The request-response communication model of the SOME/IP protocol is also used as an application layer protocol for the communication between ADAS and the gateway, while the UDP protocol is used as the transport layer protocol. The SOME/IP communication is also developed based on vsomeip.

- CAN/CANFD Bus

Vector’s VN1610 interface card supports 2 CANFD channels. The CANoe12.0 software is used to simulate the CAN/CANFD messages sent by VCU and BMS. Theoretically, the combination of VN1610 and CANoe12.0 can be used to simulate multiple ECUs, and each ECU can be used to send and receive multiple CAN/CANFD messages with different IDs.

### 5.2. Experiment Settings

A total of 3 × 5 sets of experiments were executed based on the variables of type of CAN/CANFD protocol used, the payload length of CAN/CANFD, and the cryptographic algorithms employed by the security scheme. The types of protocol used were CAN and CANFD, respectively. The payload length of the CAN message was set at 1 byte and 8 bytes. The payload length of the CANFD message was set at 64 bytes. The cryptographic algorithms used in the security schemes were AES128-CMAC, SHA256-HMAC, AES256-GCM, and Chacha20-Poly1305. The first two algorithms only provided integrity protection, while the latter two algorithms provided both integrity and confidentiality protection. Furthermore, the experiment conducted without any security scheme (None) was set as a comparison group.

We selected four evaluation indicators for comprehensively evaluating the performance of the protocol conversion on the gateway system designed; these were the CPU usage of the gateway program, the RAM usage, the overall latency, and the proportion of the effective load of the SOME/IP message sent by the gateway program. The latency of the security scheme was used as an indicator for evaluating the impact of the security scheme designed on the overall performance of the gateway system.

The difference between reading one CAN/CANFD message and reading multiple CAN/CANFD messages each time is the length of data and the latency of reading CAN/CANFD messages. The latter is mainly determined by the CAN/CANFD bus baud rate and is independent of the performance of gateway protocol conversion and security scheme. Therefore, it was specified in the program that the gateway should only convert one CAN/CANFD message at a time. The length of a single CAN/CANFD message was used to control the length of data that the gateway needed to process. The combination of VN1610 and CANoe12.0 simulated a single ECU and continuously sent CAN/CANFD messages with a fixed ID at a payload rate of 50% in order to adjust the message length conveniently. The CAN message baud rate in the experiment was set at 500 Kbps, and the CANFD message arbitration baud rate and data baud rate were set at 500 Kbps and 2 Mbps, respectively.

The experimental scheme and evaluation indicators are shown in Table 5.

**Figure 11:**
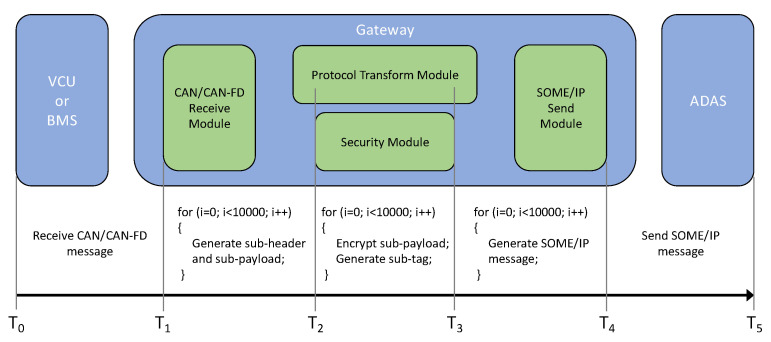
Latency indication of the designed gateway system.

The overall diagram of the latency in the designed gateway system at various stages is described in Figure 11.

(1) T0 is the time at which the SOME/IP receive module of the gateway receives the request from ADAS; that is, the time at which the gateway starts to read CAN/CANFD messages;

(2) T1 is the time at which the CAN/CANFD receive module of the gateway receives the CAN/CANFD messages;

(3) T2 is the time at which the gateway protocol conversion module generates a sub-header and sub-payload;

(4) T3 is the time at which the gateway security module calculates the sub-header and sub-payload for MAC and encrypts sub-payload to ciphertext. A total of 3 security schemes and 4 cryptographic algorithms are evaluated in this state;

(5) T4 is the time at which the SOME/IP send module of the gateway encapsulates and sends the SOME/IP message;

(6) T5 is the time at which ADAS receives the SOME/IP message sent by the gateway.

The protocol conversion and cyber security protection systems are key components of the gateway designed in this article. The latency (T4–T1) obtained from the gateway receiving the CAN/CANFD message (T1) to the gateway sent the SOME/IP message (T4) was used for the gateway performance evaluation. The portions of the gateway protocol conversion and cyber security protection were disassembled into three stages based on the execution process of the gateway program described in Section 4 during the experiment. These are generating the sub-header and the sub-payload (T2–T1), executing security algorithms (T3–T2), and encapsulating SOME/IP messages (T4–T3), respectively. The average value of each stage looping executed 10,000 times was taken as the final experimental result.

The latency of the CAN/CANFD message transmission (T1–T0) and the latency of the SOME/IP message transmission (T5–T4) from the gateway to ADAS were ignored in the performance evaluation since they were determined by the status of the CAN/CANFD bus and Ethernet. The stability of the network and the speed of the bus should have a greater impact on them. In addition, the transmission latency of CAN/CANFD and SOME/IP is one order of magnitude higher than the latency of protocol conversion and cyber security protection, which will drown the characteristics of latency drastically in the processes of protocol conversion and security protection.

### 5.3. Performance Evaluation

The proportion of effective payload is calculated by Equation (3) based on the structure of the SOME/IP frame shown in Figure 4.

(3) $$R_{original}=\frac{Length_{payload}}{Length_{header}+Length_{payload}},$$

$Length_{header}$ and $Length_{payload}$ are the data length of the SOME/IP header and the payload, respectively. $Length_{header}$ is fixed at 16 bytes and $Length_{payload}$ is a variation in which the maximum value is limited by the transport layer protocols TCP and UDP.

Cyber security protection in the process of the CAN/CANFD to SOME/IP protocol conversion is newly increased in this article. It encapsulates the payload of SOME/IP in a different way. The payload shown in Figure 8 is divided into three parts: sub-header, sub-payload, and sub-tag. The proportion of the effective load modified is calculated by Equation (4).

(4) $$R_{\mathrm{modified}}=\frac{Length_{\mathrm{sub-payload}}}{Length_{header}+Length_{\mathrm{sub-header}}+Length_{\mathrm{sub-payload}}+Length_{\mathrm{sub-tag}}},$$

$Length_{\mathrm{sub-header}}$, $Length_{\mathrm{sub-payload}}$ and, $Length_{\mathrm{sub-tag}}$ are the data lengths of SOME/IP sub-header, sub-payload and sub-tag, respectively. $Length_{\mathrm{sub-header}}$ is fixed at 4 bytes and $Length_{\mathrm{sub-payload}}$ is a variation in which the maximum value is limited by the transport layer protocols TCP and UDP. $Length_{\mathrm{sub-tag}}$ can be set at 0, 16, or 32 bytes based on the security scheme selected.

**Figure 12:**
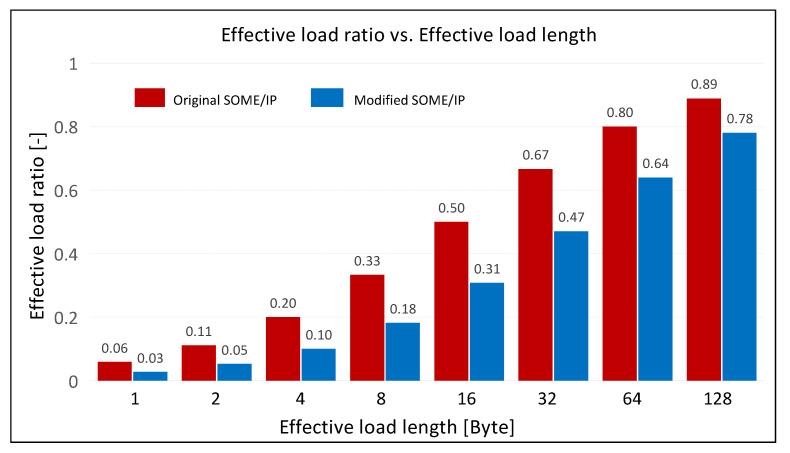
The changes in the effective load ratio with the effective load length.

The variation of the effective load ratio of SOME/IP data frame with effective load length before and after modification is shown in Figure 12. The modified SOME/IP data frame contains a sub-tag with 16 bytes. The effective load ratio before and after the modification both increase as its length increases. However, the effective load ratio of the modified SOME/IP frame accounts for lower proportions than before, and the result is more significant when the effective load length is lower after the addition of the sub-header and sub-tag fields. This consumption is valuable because the addition of the sub-tag field provides integrity for the data transmission, while the sub-header field provides a receiver with the necessary information for parsing messages.

**Figure 13:**
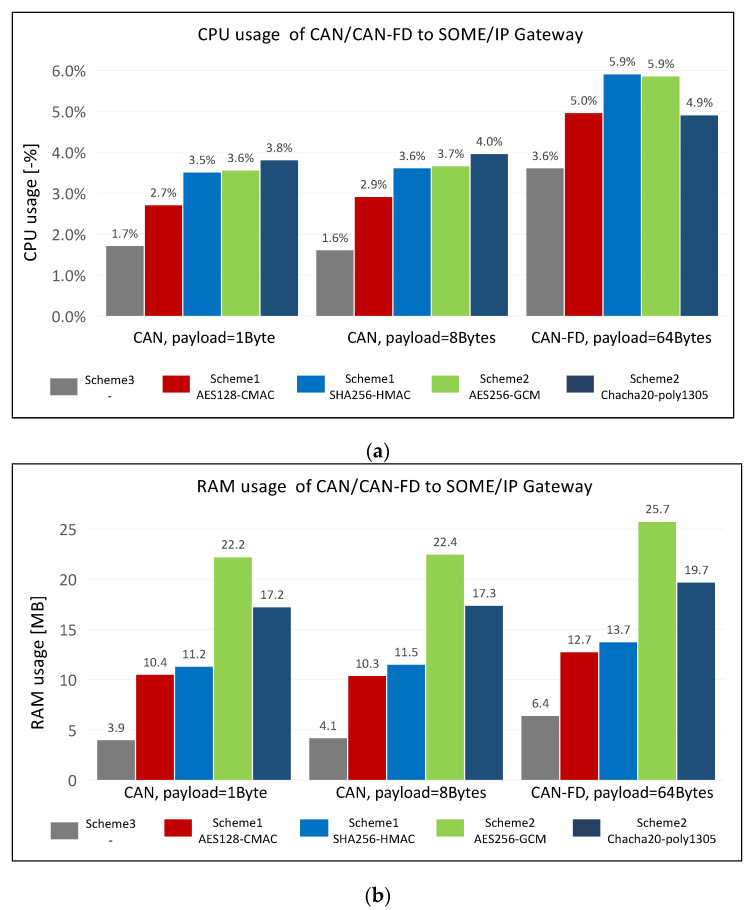
The system resource overhead of the designed gateway: (**a**) CPU usage; (**b**) RAM usage.

The CPU usage and RAM usage were measured in order to evaluate the overhead of the designed gateway in terms of system resources; the results are shown in Figure 13. The CPU and RAM usage overheads are shown in Figure 13a,b, respectively. The experimental variables are the security scheme using 4 cryptographic algorithms and the length of the CAN/CANFD payload; the experimental results are derived from the average value obtained by running the gateway program ten times continuously.

Analyzing the impact of payload length on CPU usage and RAM usage: The increase in the payload length at 7 bytes does not cause obvious variation in the CPU usage and RAM usage when comparing the experimental results with the length of 1 byte and 8 bytes, although the increase in the length of the payload must logically lead to an increase in the CPU usage and RAM usage. The experimental result gained for the payload length with 64 bytes is significantly higher than that for the payload length with 8 bytes since the increase in the length of the payload causes the amount of data that need to be converted to increase. This leads to an increase in the demand for CPU and RAM. In addition, the increase in the length of payload also means that the cryptographic algorithms require more calculations and memory space.

Analyzing the impact of security schemes on CPU usage and RAM usage: Scheme 3, without any security protection, takes up the least system resources. Scheme 2, which provides both integrity and confidentiality protection, occupies more CPU and RAM than Scheme 1, which only provides integrity based on different lengths of CAN/CANFD messages. This is because Scheme 2 requires encryption and MAC calculation, while Scheme 1 only requires MAC calculation. The overhead of AES128-CMAC is better than that of SHA256-HMAC in Scheme 1 since the NXP-S32G platform uses the Arm-A53 core, which has an AES acceleration encryption instruction set. The scheme of Chacha20-poly1305 has a slightly higher overhead with a shorter length of CAN/CANFD payload than that of AES256-GCM. However, the advantage of the Chacha20-poly1305 stream ciphers used in embedded devices becomes more obvious as the length of the CAN/CANFD payload increases, and the performance is better than that of AES256-GCM.

The designed gateway consumes a low amount of CPU and RAM in all experimental groups. The peak value of CPU usage is 5.9%. This value would increase in other devices due to the powerful performance of NXP-S32G compared to that of other embedded devices. The peak RAM usage is 25.7 MB. This value is completely acceptable for gateway devices or domain controllers that run the Linux operating system.

**Figure 14:**
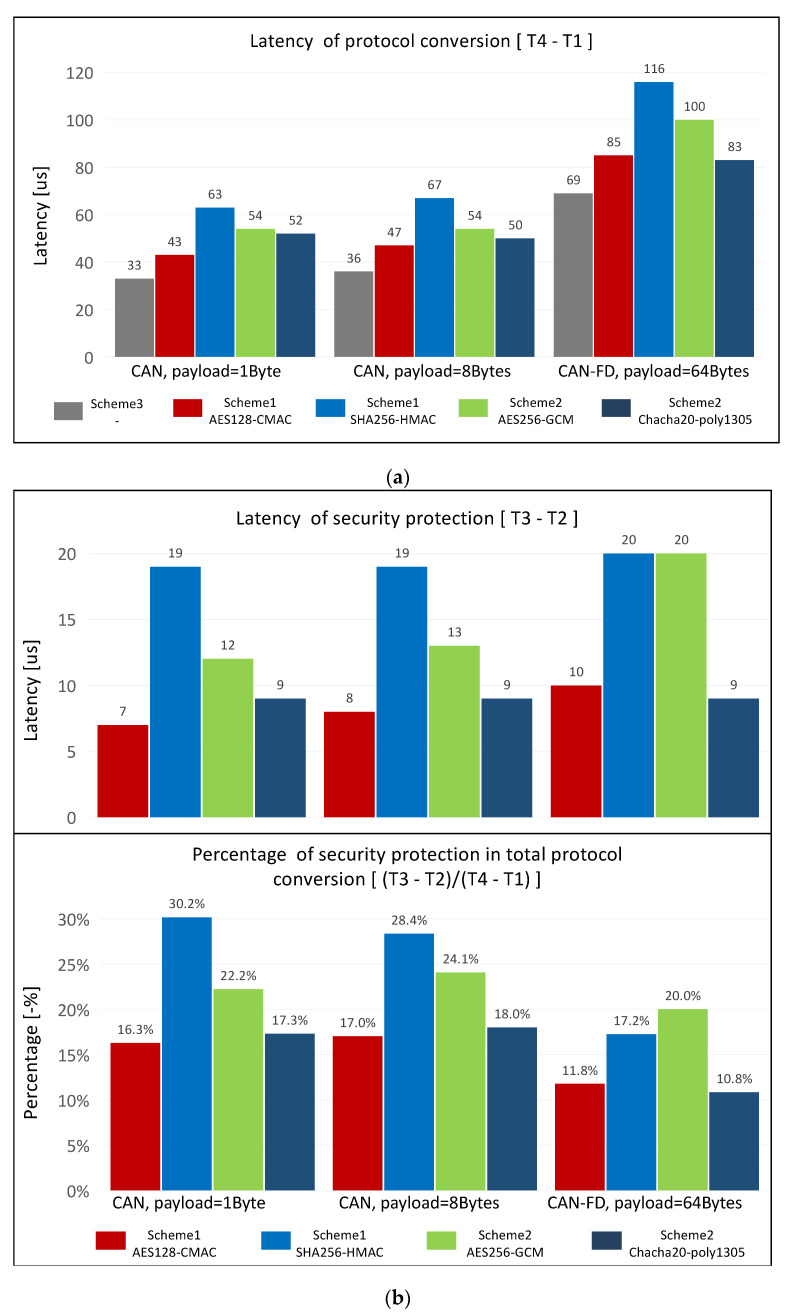
The latency characteristics of the designed gateway: (**a**) latency of the protocol conversion process; (**b**) latency of the security scheme.

The experiment tests the latency of each state between the time the gateway receives the CAN/CANFD message (T1) and the time the gateway completes the SOME/IP message transmission (T4) in order to evaluate the performance of the designed gateway. The overall latency (T4–T1) of the gateway protocol conversion is shown in Figure 14a. The latency (T3–T2) involved in security protection and its proportion in the entire protocol conversion are shown in Figure 14b. The experimental variables are security schemes with four cryptographic algorithms and the length of the CAN/CANFD payload.

Analyzing the impact of message length on protocol conversion and the latency of protection: The variation between lengths of 1 byte and 8 bytes does not cause a significant variation in the latency of T4–T1 or T3–T2, similar to the analysis of the system overhead. The latency of T3–T2 is slightly higher for the payload length of 64 bytes than for the length of 8 bytes. However, the latency of T4–T1 is significantly higher for the same condition. This shows that the increase in latency is caused by the increase in the amount of data that need to be converted and transmitted rather than the increase in the amount of data that need to be protected.

Analyzing the impact of the cryptographic algorithms used in the security scheme on the latency of protection: We focused on analyzing Scheme 1 and Scheme 2 due to Scheme 3 having the lowest latency. The two algorithms in Scheme 2 both perform encryption and MAC calculation. The latency is higher than that gained using the AES128-CMAC algorithm in Scheme 1, but its latency is lower than that gained using the SHA256-HMAC algorithm in Scheme 1. Mostly, this is because of the built-in AES acceleration instruction set in the Arm-A53 core and the excellent performance of the Chacha20-poly1305 stream cipher in the embedded devices. In addition, consistent with the experimental results of the system overhead, the performance of Chacha20-poly1305 in terms of latency is also better than that of AES256-GCM. Its latency is even close to or better than the AES128-CMAC algorithm in the condition of the high length of the CAN/CANFD payload.

Analyzing the proportion of the protection latency in the total protocol conversion: In all experimental groups, excluding the SHA256-HMAC algorithm in Scheme 1, the proportion of latency is less than 25%. Such an overhead is worthwhile since these security schemes provide integrity and confidentiality protection for the routing process. The gateway system designed in this paper contains three different levels of security protection schemes. Scheme 3, which disables security protection, could be selected when the security factors are not required during transmission.

The consumption of latency in the gateway system protocol designed is maintained at a low level, and its peak value is 116 μs. The latency accounts for a relatively low percentage compared with the Ethernet data transmission with latency in hundreds of microseconds or even milliseconds. This shows that the gateway system designed just increases a few latencies to the data transmission process while completing protocol conversion and data transmission security protection.

In the security protection scheme of Scheme 1, we recommend the use of the AES128-CMAC algorithm, which provides the same security strength (128 bit) and integrity as SHA256-HMAC and shows a lower overhead of system resources and latency based on the AES acceleration encryption instruction set. The Chacha20-poly1305 algorithm is recommended for use in Scheme 2 since the stream cipher mode is suitable for embedded and mobile devices. It performs better than the AES256-GCM algorithm while providing the same security strength (256 bit).

### 5.4. Consideration

**Figure 15:**
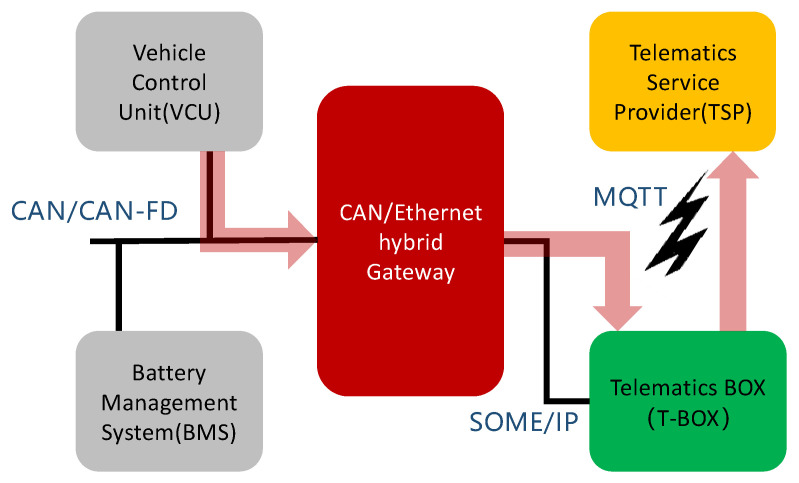
Application case of CAN/CANFD to SOME/IP.

Considering the business scenario shown in Figure 15, the vehicle data have to be reported to the cloud service platform (TSP) via a Telematics Box (T-BOX). The vehicle data are created by BMS and VCU, and the TSP is operated via a vehicle manufacturer or battery supplier. At this time, BMS and VCU need to transmit multiple frames of CAN/CANFD data to T-BOX. Considering the fact that the payload field of SOME/IP is significantly larger than that of CAN2.0 or CANFD, it is possible to encapsulate the effective information carried in several frames or even dozens of CAN/CANFD messages into one frame of SOME/IP message. On the one hand, it is necessary to add information such as the number of CAN/CANFD messages in the sub-header field of SOME/IP. On the other hand, it is necessary to organize the information stored in the sub-payload reasonably; for example, the sub-payload is further divided into multiple fields, and each field stores the effective information of one CAN/CANFD message.

In addition, the designed CAN/CANFD to SOME/IP gateway introduces a cryptographic-based security mechanism that can provide integrity and confidentiality protection, but it is not enough to prevent all attacks by hackers, such as DoS attacks. Therefore, using passive security protection technology based on cryptography can also cooperate with the active security protection technology based on intrusion detection and prevention systems (IDPS) to realize the active detection of the vehicle security status and provide more comprehensive security protection.

## 6. Conclusions

A CAN/CANFD to SOME/IP gateway system is proposed and implemented in this paper. Three security schemes with different security strengths are embedded in the routing process. The CAN/CANFD message transfer process is constructed based on a CAN socket, and the SOME/IP message send or receive processes are developed based on vsomeip. The security scheme is implemented based on the MAC algorithm (AES128-CMAC, SHA256-HMAC) and the AEAD algorithm (AES256-GCM, Chacha20-poly1305), respectively. The former only provides integrity protection for the protocol conversion process and the message transmission process, while the latter provides integrity and confidentiality protection at the same time. In this paper, we built an experimental platform based on IMX6ULL, VN1610, and the service gateway SOC S32G released in 2020 by NXP with the experimental variables of the types of CAN/CANFD protocol used, the payload size of CAN/CANFD, and the cryptographic algorithms employed by the security scheme. Four evaluation indicators were used for evaluating the performance of the designed gateway system; these are the CPU usage, the RAM usage, the overall latency, and the effective load ratio of the SOME/IP message sent by the gateway program. The main experimental results are as follows:

1. The consumption of system resources in the designed gateway system can be afforded conveniently by devices running the Linux operating system. In the experimental groups, the CPU usage of the gateway is less than 5% in most working conditions, and the RAM usage is less than 20 MB;
2. The gateway system designed just increases a few latencies to the data transmission process while completing protocol conversion and data transmission security protection. In the experimental groups, the latency of the process in the gateway system protocol conversion is less than 100 us under most conditions;
3. The proportion of latency is less than 25% for the security schemes of the gateway system designed. Such an overhead is worthwhile since these security schemes provide integrity protection and confidentiality protection for the routing process;
4. We recommend the use of the AES128-CMAC algorithm in scenarios that only need integrity protection. The performance obtained using this algorithm is significantly better than that obtained when using the SHA256-HMAC algorithm based on the AES acceleration encryption instruction set. We recommend the use of the Chacha20-poly1305 algorithm in scenarios that require both integrity and confidentiality protection.

Compared with its predecessors, the gateway system designed in this paper implements three different levels of cyber security protection scheme based on the MAC and AEAD algorithms while completing the CAN/CANFD to SOME/IP protocol conversion. It provides integrity and confidentiality protection for the process of protocol conversion and data transmission and can be modified by developers based on the application scenario required. Furthermore, the communication between the gateway and the domain controllers follows the request-response communication model of the SOME/IP protocol, which is a promising automotive SOA middleware. The proposed method has a satisfactory performance in terms of security, delivering a potential solution for current online gateway systems or future network vehicles. With the rapid development of autonomous vehicles, this high-security gateway system will be promoted more and will be used in more applications.

## Figures and Tables

**Table 1:** The contributions of this work compared to the state of the art.

Author	Verification Platform	Support Protocols	Security Mechanism
Trong [4,5]	XC7Z020, 100 MHz, Xilinx	FlexRay, Ethernet (custom)	Integrity
Jin [6,7]	TC397, 300 MHz, AURIX	CAN/CANFD, Ethernet (IEEE 1722)	Integrity
Kim [8]	MPC5668, 116 MHz, NXP	CAN, FlexRay, Ethernet (custom)	None
Shreejith [9]	ZC70x, 200 MHz, Xilinx	FlexRay, Ethernet (custom)	None
Lee [10]	TC275, 200 MHz, Infineon	FlexRay, Ethernet (custom)	None
Yadav [11]	Unspecified	CAN, Ethernet (custom)	None
Jo [12]	CANoe	CAN, CANFD, Ethernet (custom)	None
This paper	S32G, 1000 MHz, NXP	CAN/CANFD, Ethernet (SOME/IP)	Integrity, confidentiality

**Table 2:** Application of the MAC and AEAD algorithms.

Security Scheme	Algorithm Type	Security Mechanism	Algorithm	Security Strength
Scheme 1	MAC	Integrity	AES128-CMAC	128 bit
			SHA256-HMAC	128 bit
Scheme 2	AEAD	Integrity, confidentiality	AES256-GCM	256 bit
			Chacha20-Poly1305	256 bit

**Table 3:** Values of sub-header fields.

Security Scheme	Protocol Type ID	Security Level ID	Crypto Algorithms ID	MAC Length
Scheme 1, AES128-CMAC	0 for CAN2.0, 1 for CANFD	0	0	0 for 128 bit, 1 for 256 bit
Scheme 1, SHA256-HMAC		0	1	
Scheme 2, AES256-GCM		1	0	
Scheme 2, Chacha20-Poly1305		1	1	
Scheme 3		2	0	

**Table 4:** Main components of the experimental environment.

Proposed Architectures	Implementation Platform	Frequency	Baud Rate
Gateway	NXP-S32G-274evb	A53, 1000 MHz M7, 400 MHz	CAN, 500 Kbps CANFD, 500 Kbps + 2 Mbps Ethernet, 1000 Mbps
ADAS	NXP-IMX6ULL-alpha	A7, 800 MHz	Ethernet, 100 Mbps
VCU	VN1610 + CANoe12.0	-	CAN, 500 Kbps CANFD, 500 Kbps + 2 Mbps

**Table 5:** Experiments settings and evaluation metrics.

Experimental Variables			Evaluation Metrics	
CAN or CANFD	Payload size	Security algorithm	For protocol conversion	For security scheme
CAN	1 byte	AES128-CMAC	Effective load radio CPU usage RAM usage Latency	Latency
		SHA256-HMAC		
		AES256-GCM		
		Chacha20-poly1305		
		None		
CAN	8 bytes	AES128-CMAC		
		SHA256-HMAC		
		AES256-GCM		
		Chacha20-poly1305		
		None		
CANFD	64 bytes	AES128-CMAC		
		SHA256-HMAC		
		AES256-GCM		
		Chacha20-poly1305		
		None		
